# Multi-Parametric Spinal Cord MRI as Potential Progression Marker in Amyotrophic Lateral Sclerosis

**DOI:** 10.1371/journal.pone.0095516

**Published:** 2014-04-22

**Authors:** Mohamed-Mounir El Mendili, Julien Cohen-Adad, Mélanie Pelegrini-Issac, Serge Rossignol, Régine Morizot-Koutlidis, Véronique Marchand-Pauvert, Caroline Iglesias, Sina Sangari, Rose Katz, Stéphane Lehericy, Habib Benali, Pierre-François Pradat

**Affiliations:** 1 Sorbonne Universités, UPMC Univ Paris 06, UM CR 2, Laboratoire d’Imagerie Biomédicale, Paris, Île-de-France, France; 2 CNRS, UMR 7371, Laboratoire d’Imagerie Biomédicale, Paris, Île-de-France, France; 3 INSERM, U 1146, Laboratoire d’Imagerie Biomédicale, Paris, Île-de-France, France; 4 Ecole Polytechnique de Montréal, Département de Génie Électrique, Montréal, Québec, Canada; 5 Université de Montréal, GRSNC, Faculty de Médecine, Montréal, Québec, Canada; 6 AP-HP, Groupe Hospitalier Pitié-Salpêtrière, Département d’Explorations Fonctionnelles Neurologiques, Paris, Île-de-France, France; 7 Inserm U975, UPMC Univ Paris 6, UMR-S975, CNRS UMR7225, Centre de recherche de l’Institut du Cerveau et de la Moelle épinière – CRICM, Centre de Neuroimagerie de Recherche – CENIR, Paris, Île-de-France, France; 8 APHP, Groupe Hospitalier Pitié-Salpêtrière, Service de Neuroradiologie, Paris, Île-de-France, France; 9 APHP, Groupe Hospitalier Pitié-Salpêtrière, Département des Maladies du système Nerveux, Paris, Île-de-France, France; Inserm, France

## Abstract

**Objective:**

To evaluate multimodal MRI of the spinal cord in predicting disease progression and one-year clinical status in amyotrophic lateral sclerosis (ALS) patients.

**Materials and Methods:**

After a first MRI (MRI_1_), 29 ALS patients were clinically followed during 12 months; 14/29 patients underwent a second MRI (MRI_2_) at 11±3 months. Cross-sectional area (CSA) that has been shown to be a marker of lower motor neuron degeneration was measured in cervical and upper thoracic spinal cord from T2-weighted images. Fractional anisotropy (FA), axial/radial/mean diffusivities (λ_⊥_, λ_//_, MD) and magnetization transfer ratio (MTR) were measured within the lateral corticospinal tract in the cervical region. Imaging metrics were compared with clinical scales: Revised ALS Functional Rating Scale (ALSFRS-R) and manual muscle testing (MMT) score.

**Results:**

At MRI_1_, CSA correlated significantly (*P*<0.05) with MMT and arm ALSFRS-R scores. FA correlated significantly with leg ALFSRS-R scores. One year after MRI_1_, CSA predicted (*P*<0.01) arm ALSFSR-R subscore and FA predicted (*P*<0.01) leg ALSFRS-R subscore. From MRI_1_ to MRI_2_, significant changes (*P*<0.01) were detected for CSA and MTR. CSA rate of change (i.e. atrophy) highly correlated (*P*<0.01) with arm ALSFRS-R and arm MMT subscores rate of change.

**Conclusion:**

Atrophy and DTI metrics predicted ALS disease progression. Cord atrophy was a better biomarker of disease progression than diffusion and MTR. Our study suggests that multimodal MRI could provide surrogate markers of ALS that may help monitoring the effect of disease-modifying drugs.

## Introduction

Amyotrophic Lateral Sclerosis (ALS) is the most frequent motor neuron disease characterized by degeneration of both upper and lower motor neurons. Disease progression is characterized by worsening of weakness and physical disability with a median survival ranging from 2.5 to 3 years [1], [2]. There is no curative treatment and the only available neuroprotective drug is riluzole that showed only a modest effect on survival but no evidence of effect on the progression of disability [3]. There is an unmet need for biomarkers in amyotrophic lateral sclerosis, not only for better diagnosis but also to assess disease progression and the effect of disease-modifying drugs in clinical trials (surrogate markers) [4], [5], [6]. There is an increasing body of knowledge suggesting that new neuroimaging approaches, including diffusion tensor imaging (DTI) and magnetization transfer imaging, may provide promising biomarkers in ALS [7]–[10].

MRI studies in ALS mostly investigated neurodegenerative changes in the brain and thus only investigated upper motor neuron involvement [11]–[15]. Spinal cord MRI has the advantage of investigating the two motor system components that are involved in ALS, i.e the lower motor neuron (via gray matter atrophy) and upper motor neuron (via degeneration of corticospinal tract). However, spinal cord imaging has technical limitations due to the small diameter of the spinal cord, physiological motions (respiratory and cardiac movements) and susceptibility artifacts [16], [17], [18]. Recently, progress in neuroimaging protocols using multi-parametric MRI approaches (DTI, MT and atrophy measurement) allowed to detect abnormalities in the cervical cord of ALS patients [10], [19]–[22], some metrics being correlated with upper or lower motor neuron involvement as assessed by transcranial magnetic stimulation (TMS) results [10]. Two transversal MRI studies showed a correlation between CSA and clinical disability [10], [22]. For MRI metrics to be used as surrogate marker of neurodegeneration, longitudinal studies are mandatory to determine whether these metrics are sensitive to changes over time and correlate with clinical progression. To address this issue we performed a spinal cord MRI longitudinal study in a series of ALS patients.

## Materials and Methods

### Subjects

Twenty-nine patients with probable or definite ALS were enrolled in the study and underwent a baseline spinal MRI examination (MRI_1_). A follow-up MRI (MRI_2_) was performed in 14 of these patients after a mean delay of 11.1 months (±2.7 months). Follow-up MRI was not feasible in other patients due to orthopnea (n = 9), worsening of the general condition rendering transportation and MRI examination impossible (n = 1), loss of follow-up (n = 2), death (n = 2) and refusal (n = 1). The local Ethics Committee of our institution approved all experimental procedures (Paris-Ile de France Ethical Committee under the 2009-A00291–56 registration number), and written informed consent was obtained from each participant. All clinical investigations have been conducted according to the principles expressed in the Declaration of Helsinki.

Revised ALS Functional Rating Scale [23] data were obtained at MRI_1_, one year after MRI_1_ in all 29 ALS patients and at MRI_2_ for the 14 ALS patients. We calculated an arm ALSFRS-R subscore pertaining to functions such as handwriting, cutting food and handling utensils, and a leg ALSFRS-R subscore (walking, climbing stairs). The patients were also scored on manual muscle testing (MMT) using the Medical Research Council score [24]. MMT was performed at baseline in all ALS patients and at one year in the subgroup of patients who had a second MRI. Seven muscles in each limb were assessed. Information about patients’ demographics and clinical disability are given in Table 1. It includes disease duration, which was defined as the delay between the onset of weakness and MRI examination. Patients were scored on the day of each MRI.

**Table 1:** Patients demographics and clinical features in the whole population and in the subgroup of patients who had a second MRI (mean ± SD).

	At baseline		At follow up		
Characteristics	Whole population	Sub-group	Sub-group	Delta	Rate of change
Gender	9 Females/20 Males	4 Females/10 Males	–	–	–
Age	53.1±9.8 years	52.6±11 years	53.5±11 years	–	–
Recruitment period	Feb 2010– Feb 2011	Feb 2010– Feb 2011	Jun 2011– Jul 2012	–	–
Disease duration	26.8±26.9 months	28.7±27.6 months	39.8±28.8 months	11.1±2.7 months	–
Site of onset	16 Upper; 11 Lower;	6 Upper; 8 Lower	–	–	–
	2 Upper+Lower				
ALSFRS					
Arm (/8)	5.00±2.25	5.71±2.30	2.93±2.89	−48.75%	−0.25 units/month
Leg (/8)	4.72±2.47	4.93±2.23	3.50±2.71	−28.99%	−0.12 units/month
Total (/48)	37.14±6.35	39.93±6.18	30.57±9.40	−21.47%	−0.76 units/month
MMT					
Arm (/70)	52.90±11.90	56.79±11.11	45±16.44	−22.14%	−1.06 units/month
Leg (/70)	59.72±17.85	61.14±16.94	53.79±17.13	−12.03%	−0.58 units/month
Total (140)	112.62±23.80	117.93±18.29	98.00±24.95	−16.90%	−1.64 units/month
TMS					
Threshold (ms)	68.01±20.82	63.65±18.46	–	–	–
Amplitude (mV)	1.92±1.38	1.63±0.97	–	–	–

### MRI Acquisition

Acquisitions were performed using a 3T MRI system (TIM Trio, Siemens Healthcare, Erlangen, Germany), with a body coil for signal excitation and a neck/spine coil for signal reception. Anatomical imaging was performed at the cervical and upper thoracic levels. DTI and magnetization transfer imaging were performed from vertebral C2 to T2 using the following protocol:

#### Anatomical data

A sagittal T2-weighted three-dimensional (3D) turbo spin echo (TSE) image with slab selective excitation was acquired. Imaging parameters were: isotropic voxel size 0.9 mm^3^; FOV = 280×280 mm^2^; 52 sagittal slices; TR = 1500 ms; TE = 120 ms; acceleration factor = 3; acquisition time ∼ 6 min.

#### Diffusion weighted-imaging

DTI data were acquired using a single shot EPI sequence with monopolar diffusion-weighting scheme. The acquisition was cardiac-gated. Eight axial slices covering C2 to T2 vertebral levels were acquired. Imaging parameters were: voxel size = 1×1×5 mm^3^; FOV = 128×128 mm^2^; TR = 700 ms; TE = 96 ms; acceleration factor = 2; b-value = 1000 s/mm^2^; 64 diffusion encoding directions; 4 averages; acquisition time ∼ 15 min.

#### Magnetization transfer

3D gradient echo images with slab-selective excitation were acquired with and without magnetization transfer (MT) saturation pulse (Gaussian envelope, duration 9984 µs, frequency offset 1200 Hz). Imaging parameters were: voxel size = 0.9×0.9×2 mm^3^; FOV = 230×230 mm^2^; axial orientation with 52 slices (covering the same C2-T2 region as the DTI scans), TR = 28 ms; TE = 3.2 ms; acquisition time ∼5 min per volume.

For an exhaustive description of the MRI acquisition parameters, the reader is referred to [25].

### TMS Examination

TMS was performed within two weeks of the MRI_1_ using a MAGSTIM 200 device, delivering monophasic stimulation through a round coil (9 cm diameter). Responses of hand muscle (adductor digiti minimi) were recorded with surface electrodes using a KPnet system (Natus/Dantec, Denmark). The stimulation was first applied at the cervical level (C7-D1) to excite the root near its exit from the spinal cord [26]. The motor evoked potential amplitude was measured to assess lower motor neurons impairment. The motor evoked potential threshold was measured to assess CST degeneration [27] (Table 1).

### DATA Processing

#### Cord atrophy

Cord cross-sectional area (CSA) was measured by an experienced operator on the segmentation technic (i.e. four years experience) on the T2-TSE images in the middle of vertebral levels from C2 to T6 using the semi-automatic method that has been shown to be accurate and with a low inter-observer variability [28], [29], [30]. The plane perpendicular to the spinal cord was resampled to minimize partial volume between spinal cord and cerebrospinal fluid [31]. To increase reproducibility and accuracy of the used segmentation method, images were segmented by an experienced operator on the technic.

#### DTI

Data were corrected for motion slice-by-slice using FSL FLIRT [32] with three degrees of freedom (Tx, Ty, Rz). Diffusion metrics were estimated voxel-wise using FSL DTIFIT: fractional anisotropy (FA), radial diffusivity (λ_⊥_), axial diffusivity (λ_//_) and mean diffusivity (MD).

#### Magnetization transfer

Gradient echo volumes with and without magnetization transfer pulse were coregistered using FSL FNIRT non-linear algorithm. Magnetization transfer ratio (MTR) was computed voxel-wise following the equation 100 × ((S_0_ − S_MT_)/S_0_), where S_0_ and S_MT_ represent the signal without and with the magnetization transfer pulse, respectively.

#### ROI-based analysis

**Figure 1:**
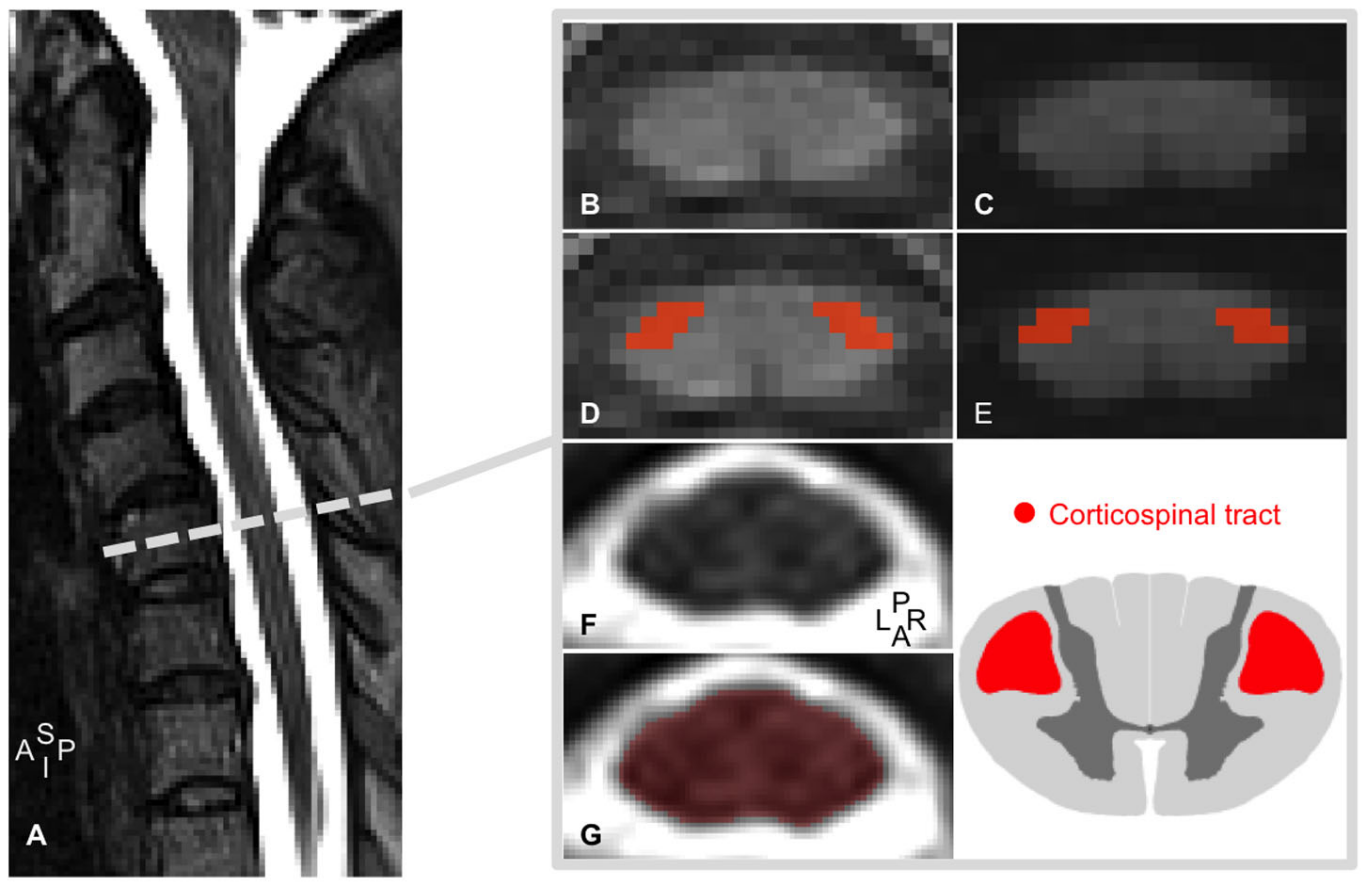
Regions of interest definition. (A) T2-weighted MRI mid-sagittal slice for an ALS patient. (B,C) T1-weighted data for MTR (B) and mean diffusion-weighted data for DTI (C). (D,E) Defined anatomical ROIs. ROIs were selected in the dorso-lateral (red) aspect of the spinal cord to include most of the CST. (F,G) T2-wheighted data before (F) and after (G) cord outlining. A, anterior, I, inferior; L, left; R, right; P, posterior; S, Superior.

The lateral portion of the cord was delineated manually and using geometry-based information by an experienced operator on segmentation. To minimize bias, these regions of interest (ROIs) were defined on the mean diffusion weighted images (for DTI analysis) and on the 3D gradient-echo T1-weighted image (for MT analysis) (Figure 1). This method has been shown to be sensitive enough to discriminate changes between sensory (posterior) and motor (lateral) tracks in the context of ALS [10] and spinal cord injury [25]. For an exhaustive description of ROIs definition, the reader is referred to [25].

### Statistics

Statistical analysis was performed using Matlab (The Mathworks Inc, MA, USA).

#### Correlations of MRI metrics with clinical disability at baseline

Stepwise linear regression was used to find the best predictor of clinical disability at MRI_1_ in all 29 patients. Dependent variables were: ALSFRS-R (total, arm subscore and leg subscore) and MMT. Predictors were: gender, age, site onset, disease duration, FA, λ_⊥_, λ_//_, MD, MTR, cross-sectional area, MEP threshold and amplitude. The probability for a predictor to enter the stepwise model was based on a Fisher's test, with a *P*-value set to 0.05.

#### Prediction of clinical disability after one-year follow-up

Stepwise linear regression was used to find the best predictor of clinical disability in all 29 ALS patients one year after MRI_1_. Dependent variable was: ALSFRS-R (arm, leg and total scores) after one-year follow-up. Predictors were: gender, age, site onset, disease duration, FA, λ_⊥_, λ_//_, MD, MTR, cross-sectional area, MEP threshold and amplitude measured at baseline. The probability for a predictor to enter the stepwise model was based on a Fisher's test, with a P-value set to 0.05.

### Longitudinal MRI Study

#### Comparisons between baseline and follow-up MRI

Wilcoxon signed rank test was performed to evaluate changes in MRI metrics from MRI_1_ to MRI_2_. Correlation between changes in MRI metrics over time and progression of clinical disability. Spearman's rank correlation coefficient was used to investigate correlations between MRI metrics and rate of change of clinical disability scores, defined as the change of a variable between MRI_1_ and MR_2_ scans, divided by the time interval between the two scans.

## Results

### Correlations of MRI Metrics with Clinical Disability at Baseline

Cord CSA was the only significant predictor associated with the arm subscore of ALSFRS-R (*P* = 0.049) and MMT (*P* = 0.019 for arm and 0.04 for leg) scales. FA measure in the lateral segment of the spinal cord was the only predictor of leg subscore (*P* = 3.10^−4^) and total score (*P* = 0.039) of the ALSFRS-R. Results of stepwise linear regression analysis are summarized in Table 2 (Bonferroni non corrected, significance level *alpha* = 0.0056).

**Table 2:** Results of stepwise linear regressions.

	ALSFRS-R			MMT		
Predictors	Arm	Leg	Total	Arm	Leg	Total
FA	0.7198	0.0003(*)	0.0393(*)	0.7766	0.1280	0.1975
λ_⊥_	0.6664	0.4428	0.7222	0.5399	0.1439	0.5993
Cord area	0.0493(*)	0.1492	0.4142	0.0193(*)	0.0434(*)	0.8317

Dependent variables were the ALSFRS-R (arm, leg and total scores) and the MMT (arm, leg and total scores) at MRI_1_. Predictors were gender, age, disease duration, site of onset, DTI metrics (FA, λ_⊥_, λ_//_and MD), MTR, CSA, TMS measurements (MEP threshold and amplitude). Significant values are marked with (*). Only significant predictors are represented here.

### Prediction of Clinical Disability after One-year Follow-up

Results of the stepwise linear regression analysis revealed that the mean spinal cord CSA at MRI_1_ was the only predictor of the arm ALSFRS-R subscore (*P = *0.013, Table 3). FA measured at MRI_1_ was the only predictor of the leg ALSFRS-R subscore (*P = *0.002). FA, λ_⊥_ and CSA measurements predicted the total ALSFSR-R score (*P*_FA = _2.10^−4^, *P*_λ⊥ = _0.009, *P*_CSA = _0.014). There was no predictor of the decline of the arm or leg ALSFRS-R subscores (*P*>0.05) (Bonferroni non corrected, significance level *alpha* = 0.0056).

**Table 3:** Results of stepwise linear regressions.

	ALSFSR-R		
Predictors	Arm	Leg	Total
FA	0.5342	0.0022(*)	0.0002(*)
λ_⊥_	0.6667	0.1377	0,0093(*)
Cord area	0.0129(*)	0.7775	0.0140(*)

Dependent variables were the ALSFRS-R (arm, leg and total scores) one year after MRI_1_. Predictors were gender, age, disease duration, site of onset, DTI metrics (FA, λ_⊥_, λ_//_and MD), MTR, CSA, TMS measurements (MEP threshold and amplitude). Significant values are marked with (*). Only significant predictors are represented here.

### Comparisons between Baseline and Follow-up MRI

MRI metrics for MRI_1_ and MRI_2_ in ALS patients are indicated in Table 4.

**Table 4:** MRI metrics at baseline and follow-up in ALS patients (mean ± SD).

MRI metrics	Baseline	Follow-up	*P*-value	Delta	Rate of change
FA	0.523±0.054	0.512±0.048	0.168	–	–
λ_⊥_ (×10^−3^ mm^2^/s)	0.740±0.128	0.782±1.113	0.127	–	–
λ_//_ (×10^−3^ mm^2^/s)	1.740±0.117	1.679±0.102	0.052	_	–
MD (×10^−3^ mm^2^/s)	1.051±0.115	1.087±0.098	0.068	_	–
MTR	33.22±2.51	30.00±3.15	0.0029(*)	−9.68%	−0.33 units/month
Cord area (mm^2^)	59.7±6.7	58.1±6.9	0.00085(*)	−2,73%	−0.14 mm^2^/month

Significant differences are marked with (*).

**Figure 2:**
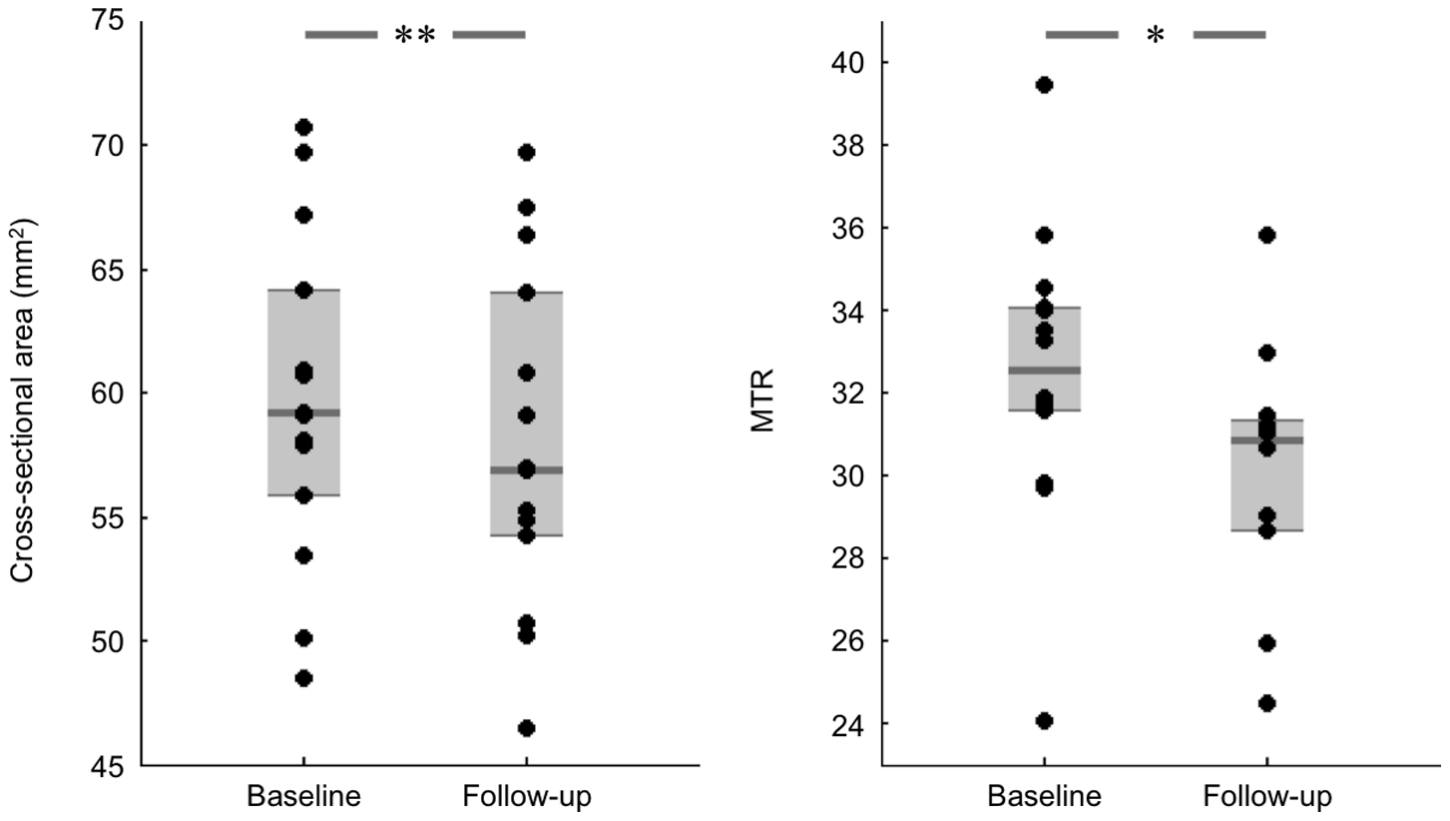
Comparisons between ALS patients at baseline and follow-up for individual cross-sectional area and MTR in the dorso-lateral aspect of the spinal cord that includes mostly the corticospinal tract. Group differences were assessed using Wilcoxon signed rank test. Levels of significance are indicated as: *P<0.01, **P<0.001.

The cord CSA decreased in all patients, except in one case, by a mean of 0.14 mm^2^/month (*P* = 9.10^−4^). Only one patient did not show a decrease in spinal cord area (minor increase of 0.04 mm^2^/month), within the range of sensitivity of the segmentation method [30]. Furthermore, this patient had a very long disease duration (9 years) and was clinically stable between the two MRI. MTR decreased by 0.33 units/month (*P = *0.003). Figure 2 shows plots of CSA and MTR at baseline and follow-up.

No significant change was detected either for FA (*P* = 0.168), λ_⊥_ *(P = *0.127), λ_//_(*P* = 0.052) or for MD (*P* = 0.068) (Bonferroni corrected, significance level *alpha* = 0.008).

### Correlation between Changes in MRI Metrics Over Time and Progression of Clinical Disability

**Figure 3:**
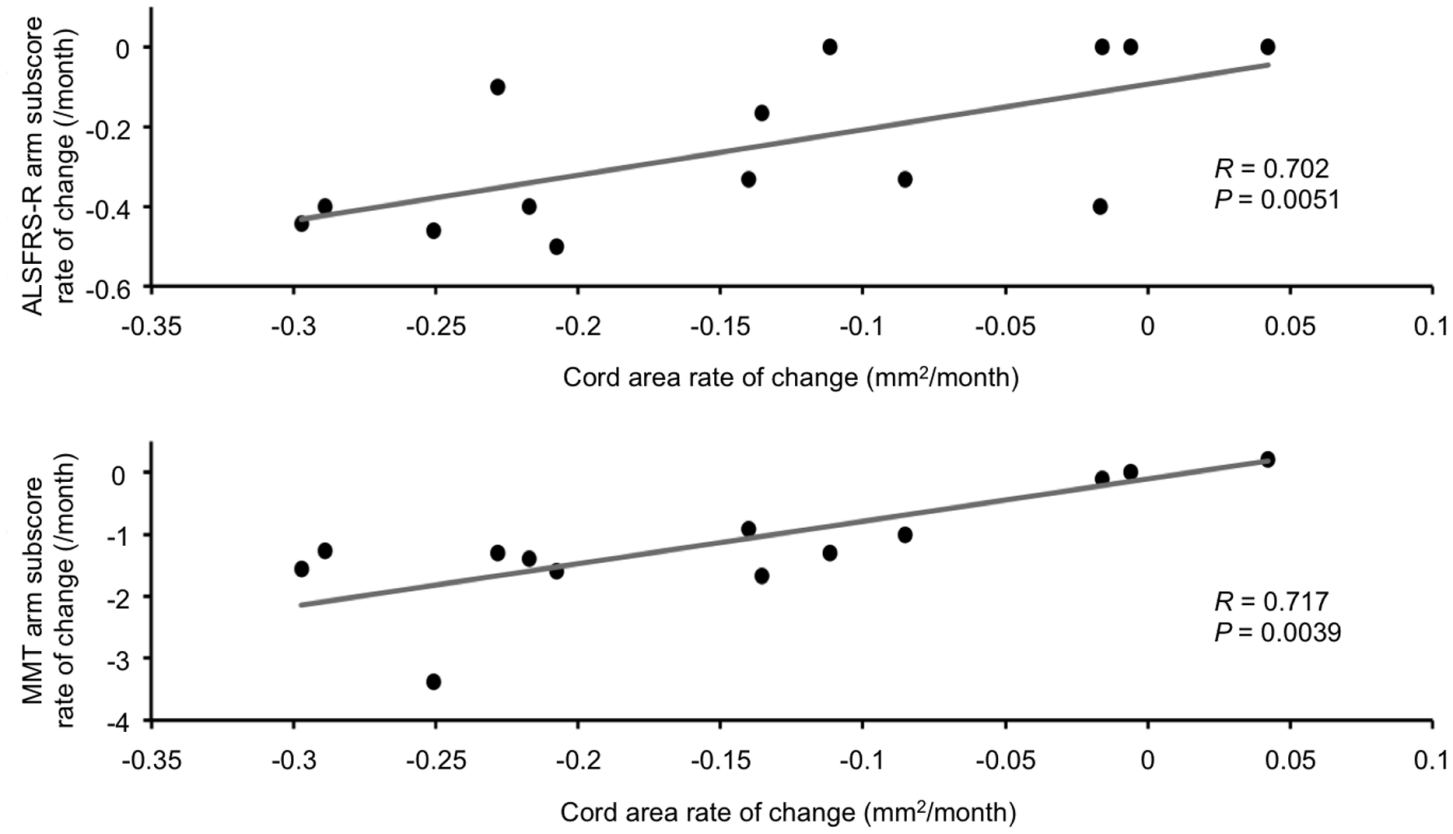
Correlations between cord area rate of change and the ALSFRS-R arm subscore rate of change and MMT arm subscore rate of change. Correlation coefficients and *P*-values are derived from Spearman's correlation.

Results of correlations are shown in Figure 3. The atrophy rate of the spinal cord area (i.e., CSA rate of change) was highly correlated with worsening in clinical functional status, the correlations being higher when considering the arm subscore of the ALSFRS-R (*R* = 0.702, *P* = 0.005) and the arm subscore of the MMT (*R* = 0.717, *P = *0.004). No correlations were detected between the atrophy rate of change and rates of changes in the leg subscore of the ALSFRS-R (*R* = 0.376, *P* = 0.185), the total ALSFSR-R (*R* = 0.481, *P* = 0.084), and the leg subscores of the MMT (*R* = 0.153, *P* = 0.601).

No correlations were detected between the rate of change in MTR and the rates of changes in the arm ALSFRS-R (*R* = 0.037, *P* = 0.914), leg ALSFRS-R (*R* = −0.432, *P* = 0.184), arm MMT (*R* = −0.059, *P* = 0.863) and leg MMT (*R* = 0.009, *P* = 0.979) subscores (Bonferroni corrected, significance level *alpha* = 0.025).

## Discussion

Results show that spinal cord atrophy and MTR are sensitive the progression of the tissue neurodegenerative process in the spinal cord in ALS patients. The cord area at baseline was predictive of the functional ability of the arms after a one-year follow-up duration. Between MRI_1_ and MRI_2_, MTR decreased significantly in the lateral segments of the spinal cord by 0.33 units/month and the cross sectional area decreased significantly by 0.14 mm^2^/month. Furthermore, the atrophy rate of change was strongly correlated with the rates of change in the arm ALSFRS-R and MMT subscores.

Significant decrease in cord area in ALS patients followed longitudinally was previously reported [20], although no correlation with the ALSFRS score was found. Several factors may explain this lack of correlation in the study of Agosta et al. First, the lower field strength (1.5T versus 3T) might have been associated with lower resolution and/or lower signal-to-noise ratio, yielding less precision in delineating the spinal cord. Second, the present study includes a larger rostro-caudal portion of the spinal cord, which may have increased the sensitivity to detect motor neuron degeneration affecting the ALSFRS score. Third, the follow-up period was shorter (9 versus 11 months in the present study) and patients showed a slower ALSFRS decline (0.5 versus 0.8 in the present study). Finally, variability in the population studied, given the small sample sizes, may also account for the differences. Among our cohort of 29 patients, a follow-up MRI was only feasible in 14 patients. This relatively high level of drop-out rate (52%) is common in longitudinal studies in ALS (from 27% to 75%, depending on the duration of the follow-up) [20], [33]–[39]. The development of respiratory insufficiency responsible for orthopnea and severe disability are classical limitations for performing a re-scan in ALS patients.

Our result suggest that changes over time of spinal cord MRI metric may reflect to some extent the respective contribution of lower and upper motor neuron degeneration to disability. Changes of FA measured in the lateral spinal cord containing mostly the cortico-spinal tract were linked to leg disablity. These results are reminiscent of clinical observations, since symptoms due to upper motor neuron degeneration, particularly spasticity, mainly affect the lower limb. Stiffness in the lower limb, which is associated with balance and posture impairment, is a major cause of disability even in ALS patients without noticeable motor deficit in the lower limbs [40]. In our previous study, we showed that CSA was correlated with MEP amplitude, that is a TMS index of lower motor neuron involvement, and not with MEP threshold, that is a TMS index of upper motor neuron involvement [10]. So as, the observed correlations between atrophy rate of change and arm functional sub-scores decline suggest that lower motor neuron degeneration was the determinant factor of the arm disability progression. However, future study using higher resolution enabling separation between grey and white matter may provide more arguments to support these assumptions.

It is possible that cord atrophy partly contributed to the longitudinal decrease in MTR due to partial volume effects with adjacent CSF and gray matter, which may have lowered the apparent MTR calculation from averaging effects. However, several arguments support that partial volume effect minimally contributed to the detected effect. Firstly, although cord area was significantly smaller at follow-up, the variation was about 1.6 mm^2^, which corresponds to a change in radius of less than five microns (using circular approximation of the cord). Given that pixel size is 1×1 mm^2^, the partial volume effect resulting from the coarse grid-based definition of the ROIs–which only encompass 10–15 voxels over the corticospinal region–dominates in comparison to the change in cord diameter. In other words, the variability introduced by the coarse sampling of MRI markers is orders of magnitude higher than the systematic bias introduced by a small change in the cord size. Secondly, during delineation of the cord we opted for a conservative approach, by leaving out pixels that were at the cord/CSF or white matter/gray matter interfaces. Although this approach has flaws, this is the currently adopted approach for quantifying MRI metrics in the spinal cord. Thirdly, if it were a pure partial volume effect, differences would also be observed in the diffusion metrics submitted to the same processing (spatial resolution was almost the same for DTI and MTR). However, neither significant difference nor trends were observed for diffusion metrics between baseline and follow-up. Although previous studies introduced atrophy as a confound for isolating the effect of a dependent variable [41], it is often a delicate process due to the high correlation between atrophy and other MRI metrics (not due to partial volume effects, but due to degenerative processes).

## Conclusion

In conclusion, we demonstrated a significant relationship between atrophy rate and disease progression in our cohort of ALS patients. In addition, our results suggest that spinal cord cross-sectional area and MTR are more reliable markers of longitudinal neurodegeneration in the spinal cord compared to DTI metrics such as FA or radial diffusivity. However the somewhat lower sensitivity of diffusion MRI might be overcome thanks to the current improvements in hardware, acquisition and processing techniques. Further longitudinal studies in a larger population are needed to validate atrophy rate of the spinal cord as a valid surrogate marker of disease progression in ALS.
